# Addressing barriers to primary health-care services for noncommunicable diseases in the African Region

**DOI:** 10.2471/BLT.20.271239

**Published:** 2020-10-05

**Authors:** Azeb Tesema, Rohina Joshi, Seye Abimbola, Whenayon Simeon Ajisegiri, Padmanesan Narasimhan, David Peiris

**Affiliations:** a The George Institute for Global Health, University of New South Wales (UNSW), Level 5, 1 King Street Newtown NSW 2042, Sydney, Australia.; b School of Public Health, University of Sydney, Sydney, Australia.; c School of Public Health and Community Medicine, UNSW, Sydney, Australia.

The World Health Organization (WHO) *Package of essential noncommunicable disease interventions* (known as WHO PEN) was designed for the prevention, early detection, treatment and care of diabetes, cancer, chronic respiratory diseases, cardiovascular diseases and associated risk factors. The package is a well-established, cost-effective and action-oriented strategy and the recommended interventions can be considered first-line options for improving the integration of management of noncommunicable diseases into primary health care.^1^

These interventions are generally suitable for implementation in low- and middle-income countries. The global experience of the adoption of the WHO package and the resulting health impacts are largely encouraging, with several countries having successfully improved the management of noncommunicable diseases in primary health-care settings.^1^^,^^2^ However, in the African Region, many countries are yet to adopt the WHO package and other related noncommunicable disease interventions. Many of the countries that have adopted the interventions are yet to implement them on a national scale.^3^ Of the 35 countries in WHO African Region with multisectoral noncommunicable disease action plans, only four (Benin, Eritrea, South Africa and Togo) report having a nationally integrated package of interventions.^3^ An additional eight other countries (Botswana, Burkina Faso, Côte d’Ivoire, Eswatini, Ethiopia, Guinea, Lesotho, Malawi and Sierra Leone) have partially implemented the WHO package into their national health system.^3^ A key question, therefore, is how national policies and implementation plans can be accelerated to maximize uptake of the WHO package in the Africa Region.^1^

While it is beyond the scope of this paper to conduct country-specific analyses, the implementation framework for the WHO package is a useful way to identify barriers to uptake of recommended interventions.^1^ Recent studies in Botswana, Ghana and Uganda identified several barriers that align with the framework of the package, including a lack of simplified protocols or guidelines;^4^ inadequate staffing, training and supervision of primary health-care workers;^5^^,^^6^ limited availability of health facility infrastructure; limited supply of essential medicines and diagnostic technologies;^4^^–^^6^ and inadequate data for monitoring and evaluation of interventions for noncommunicable diseases.^4^^,^^6^

Taking into consideration these barriers, and based on our collective experience of working on noncommunicable diseases in primary health care, we draw attention to where action is needed to accelerate progress and improve uptake of the WHO package. We have identified seven priority areas: (i) political commitment; (ii) service re-design; (iii) workforce capacity strengthening; (iv) treatment protocols; (v) resource gap analysis; (vi) implementation at scale; and (vii) support from multilateral institutions.

## Political commitment

The involvement and political commitment of key people in government is an essential first step in ensuring early adoption and sustainability of noncommunicable disease interventions.^1^^,^^7^ Moreover, the extent to which technical guidance such as the WHO package is adapted to local contexts and capability appears to be a critical driver of its adoption. A recent systematic review of the interventions aligned to WHO’s “Best Buys” highlighted the need for noncommunicable disease interventions to be tailored to the local context and their application in specific settings.^8^ Local adaptation requires health system managers to work in close collaboration with senior politicians and bureaucrats to develop strategic plans. The experience from Cabo Verde in involving relevant stakeholders during adoption of the protocol and engagement with WHO is a good example.^9^

However, political commitment is a necessary but not sufficient condition for adapting global action plans into meaningful policy tools at the front line of care delivery. Good governance, resource mobilization, advocacy and engagement of multisectoral stakeholders, and strong monitoring and evaluation frameworks for programmes, are vital inputs to support implementation of the WHO package strategies. Institutions are fragile in many parts of the African Region; policy-makers have limited capacity in programme and policy development and there is a weak research infrastructure for monitoring and evaluation.^3^

## Service re-design

Many lessons can be learnt from success with chronic infectious disease programmes, in particular those for human immunodeficiency virus (HIV) infection, tuberculosis and malaria.^4^^,^^7^ The existence of relatively developed HIV service delivery systems in Africa can offer a unique opportunity for the integration of noncommunicable disease service delivery within primary health-care systems.^7^ The well-documented evidence of how HIV programmes stimulated service decentralization and rejuvenated primary health-care facilities is highly relevant to noncommunicable diseases.^7^ Workable facility and community-based HIV service delivery platforms (such as HIV prevention, counselling and testing for most at-risk and vulnerable populations) can be built on for delivering an integrated and comprehensive noncommunicable disease service.^7^ Training and empowerment of facility management teams could also play an important role in achieving noncommunicable disease prevention and control goals. Further to this is the need to strengthen existing noncommunicable disease units at subnational levels or to establish new ones if they are not already in place. Along with these actions, countries may need to designate dedicated officers to coordinate a noncommunicable disease programme at national and subnational levels.^1^ Experiences from other countries show that establishing an independent noncommunicable disease agency to advocate for and mobilize resources can be important in bringing the noncommunicable disease agenda to the forefront.

Compared with the well-resourced and coordinated HIV programme, however, the implementation of a noncommunicable disease programme will have its own challenges. Such challenges include lack of coordination; programme- or disease-specific national funding; inadequate numbers and low skills of primary health-care workers; and inadequate infrastructure.^7^ Strengthening the primary health-care system as a whole system, with all the necessary human, financial and political resources, is needed to sustain an integrated approach to noncommunicable disease prevention and control.

## Workforce capacity strengthening

On-the-job training and supportive supervision of primary health-care workers can improve their knowledge and performance in implementing the WHO package of noncommunicable disease interventions.^5^^,^^10^ Measures include provision of decentralized in-service training and mentoring, assisted by technologies (such as mobile devices) at the district and health-facility level. Training and assigning focal persons for noncommunicable diseases can also improve the continuity of service delivery. In addition, training and preparing community health workers to support noncommunicable disease service delivery through prevention, health promotion, registration and referral systems contributes to providing accessible, long-term community-based care in primary health-care facilities. Such an approach may be necessary in the African Region where much of the population live in hard-to-reach areas.^11^

## Treatment protocols

There is emerging evidence of success with developing contextualized guidelines involving local adaptations of clinical support tools.^12^ The Practical Approach to Care Kit is a simplified guide that has been implemented in Botswana, Ethiopia, Nigeria and South Africa.^12^ Comparisons across different countries have shown it to be useful for supporting clinical decision-making and for filling the know-how gap for primary health-care workers to provide services while strengthening the health system.^12^ This approach to developing disease-specific guides based on the context and level of disease burden and on-the-ground realities has potential to be applied across multiple noncommunicable diseases.

## Resource gap analysis

Overcoming resource gaps is important for optimizing primary health-care capacity to manage noncommunicable diseases, particularly in relation to infrastructure, information systems, equipment and medicines shortages.^5^^,^^6^ Alignment of medicine and treatment algorithms to the WHO package needs to take into consideration each country’s essential drug lists. Strengthening information management systems to support continuous surveillance of noncommunicable disease data is essential.^6^ Using HIV data systems to improve integration of noncommunicable disease data into national electronic health information systems is another way in which HIV services can support health-system strengthening efforts for noncommunicable disease management.^7^

## Implementation at scale

To establish continuous learning from these implementation strategies, it is essential to design initiatives that can be implemented and evaluated at scale. There is a lack of evidence from the African Region on the effectiveness of the WHO package of noncommunicable disease interventions and other best-buys interventions that have been implemented at scale.^8^ Studies in the African Region have focused on pilot implementation and these models have generally not been amenable to delivery at scale.^5^^,^^6^ WHO Regional Office for Africa, its partners and Member States have embarked on new initiatives (called PEN-Plus) to expand access to high-quality care for severe, chronic noncommunicable diseases.^13^

The new initiatives focus on providing integrated outpatient care to address a set of severe noncommunicable disease conditions (such as type 1 diabetes, heart failure and sickle-cell disease) by mid-level providers at first-level or district hospitals. Although this strategy focuses on higher-level facilities, the lessons learnt from its implementation have the potential to accelerate progress at primary health-care facilities through improved integration of care between the hospital and primary care sectors.^13^ The research consortium of the Global Alliance for Chronic Diseases is another example of a promising initiative to further new knowledge on the science of scale-up. Six initiatives are under way for diabetes and hypertension care in Eswatini, Kenya, South Africa, Uganda and United Republic of Tanzania.^14^

## Support from multilateral institutions

Technical support from global institutions such as WHO can be a catalyst to the uptake and implementation of noncommunicable disease interventions. The consultative forum organized by WHO Regional Office for Africa, the Member States and its partners allowed countries to share their progress and discuss strategies to address common challenges in implementing the WHO package of noncommunicable disease interventions across Africa.^13^ Beyond knowledge-sharing fora, there is also a pressing need to provide technical support to enhance national capacity. The partnership between WHO and the West African Health Organization to train national noncommunicable disease programme managers on the WHO package is a case in point that needs further strengthening.^10^

## Conclusion

Few countries in the African Region are achieving optimal implementation of the WHO package of essential noncommunicable disease interventions in their primary health-care facilities. While the context varies, lessons can be learnt from the four countries that have successfully integrated the WHO package into primary health-care service delivery. A greater emphasis is needed on identifying these success stories, applying the lessons learnt to other health system contexts, and understanding the factors needed to support implementation at scale.
